# Foods and Supplements Associated with Vitamin B_12_ Biomarkers among Vegetarian and Non-Vegetarian Participants of the Adventist Health Study-2 (AHS-2) Calibration Study

**DOI:** 10.3390/nu10060722

**Published:** 2018-06-04

**Authors:** Didit Damayanti, Karen Jaceldo-Siegl, W. Lawrence Beeson, Gary Fraser, Keiji Oda, Ella H. Haddad

**Affiliations:** 1 Loma Linda University, School of Public Health, Loma Linda, CA 92350, USA; diditdamayanti@yahoo.com (D.D.); kjaceldo@llu.edu (K.J.-S.); lbeeson@llu.edu (W.L.B.); gfraser@llu.edu (G.F.); koda@llu.edu (K.O.); 2 Politeknik Kesehatan Kemenkes Jakarta II, Jakarta 12120, Indonesia

**Keywords:** vitamin B_12_, dietary intake, vegetarian

## Abstract

To investigate the association between plasma concentration of vitamin B_12_ and B_12_ intake from supplements, fortified foods, and animal source foods among vegetarians and non-vegetarians, we conducted a cross-sectional analysis among 728 participants of the Adventist Health Study 2 (AHS-2) calibration study. The median age of participants was 58 years, 65.4% were female, and 50.3% were White. We used six 24 h dietary recalls to measure B_12_ intake, serum vitamin B_12_, and holotranscobalamin (holoTC) concentration. B_12_ supplements had a significantly positive association with plasma B_12_ among all subjects (*p* trend < 0.001), especially among vegans and lacto-ovo vegetarians (*p* trend < 0.001). Among non-users of B_12_ supplements, B_12_ intake from milk substitutes was significantly positively associated with holoTC (*p* trend < 0.004) and serum B12 (*p* trend < 0.030). In non-vegetarians, holoTC was significantly positively associated with B_12_ intake from eggs, while serum B_12_ was significantly positively associated with B_12_ intake from milk in the upper tertile compared to the lower, and B_12_ intake from meat in the middle compared to the lower tertile intake (*p* < 0.011). Supplements containing B_12_ followed by B_12_ intake from milk substitutes were significant contributors of plasma vitamin B_12_ concentration.

## 1. Introduction

The prevention of low and marginal vitamin B_12_ status is important because inadequate vitamin B_12_ can lead to serious neurologic and neuropsychiatric abnormalities among adults and the elderly, even without associated anemia. A marginal vitamin B_12_ deficiency has been shown to be associated with a higher homocysteine level and increased risk of vascular disease, which can lead to cardiovascular disease and neurological deficits [1,2,3]. For example, in a study of 549 community-dwelling individuals aged 74.8 ± 4.6 years, investigators reported that plasma vitamin B_12_ levels between 187–256.8 pmol/L predicted cognitive decline in subjects compared to higher ranges of plasma vitamin B_12_ [4]. Further, Qin Bo (2017) showed in their study that higher vitamin B_12_, B_6_, folate, and niacin intake throughout young adulthood to elderly result in better cognitive assessment [5].

It is known that diet and malabsorption can cause low concentrations of plasma vitamin B_12_ among vegetarians and non-vegetarians. Besides malabsorption, adherence to a vegan diet is a factor that may contribute to inadequate vitamin B_12_ intake [1]. A review study showed low serum vitamin B_12_ and elevated homocysteine concentrations among vegetarian children, pregnant women, adults, and the elderly, particularly among vegans [2].

Considering the seriousness of the manifestations of low and marginal plasma vitamin B_12_ and factors contributing to this condition, it is necessary to examine reliable dietary sources of vitamin B_12_ that relate to plasma vitamin B_12_ concentration. There have been discrepant results about the relationship between dietary sources of vitamin B_12_ and vitamin B_12_ status. In a study that labeled foods with radioactive vitamin B_12_ among healthy individuals, Watanabe [6] found that meat, fish, and chicken were major sources of vitamin B_12_, due to its bioavailability in these foods. Tucker and colleagues [7] found intake of supplements, fortified cereals, and milk to be the sources of vitamin B_12_ that were significantly associated with plasma vitamin B_12_ among 2999 healthy individuals aged 26–83 years in the U.S. Vogiatzoglou and colleagues [8] found milk, other dairy products, and fish to be significantly positively associated with plasma vitamin B_12_ concentration among 5937 healthy individual aged 47–49 years and 71–74 years in Norway. More recently, Brouwer-Brolsma and colleagues [9] found that intake of vitamin B_12_ from dairy, meat, followed by fish and shellfish were significantly associated with serum vitamin B_12_, whereas and vitamin B_12_ intake from eggs was not significantly associated with serum vitamin B_12_. The discrepancy of study results that relate sources of vitamin B_12_ to plasma vitamin B_12_ might be due to differences in the bioavailability of vitamin B_12_ from various sources among different populations.

In the present study, we conducted descriptive analysis to determine dietary factors that are associated with plasma concentrations of vitamin B_12_, and to examine how these factors differ in vegetarians and non-vegetarians, and also among vitamin B_12_ supplement users and non-users. We used data from The Adventist Health Study-2, a cohort characterized by its large number of non-smokers, non-alcohol users, and varied eating behaviors ranging from vegan (8.0%), lacto-ovo-vegetarian (28.2%), pesco-vegetarian (9.9%), and semi-vegetarian (5.6%) to non-vegetarian (48.3%) [10]. These characteristics provide a unique opportunity to identify the dietary sources of vitamin B_12_, specifically, vitamin B_12_ intake from supplements, fortified foods, and animal source foods (that include meat, fish, milk, and eggs) in a health-conscious population.

## 2. Materials and Methods

### 2.1. Subjects

A cross-sectional data analysis was conducted using the Adventist Health Study-2 (AHS-2) calibration study [11], the participants of which were a representative sample of the parent AHS-2 cohort. Briefly, calibration subjects (*n* = 1011) were randomly selected by church and then within the church by gender and age from among the 96,000 participants of the AHS-2. Participants were required to attend a clinic where anthropometric data were measured and blood samples collected. The study was approved by the Institutional Review Board of Loma Linda University [10].

The inclusion criteria for this present study were subjects who had serum holotranscobalamin (holoTC) and serum vitamin B_12_ data, six 24 h dietary recalls, and dietary pattern data. Participants who had missing data (*n* = 238) from these variables were excluded from analysis. We also excluded 15 subjects who were considered outliers due to their intake of vitamin B_12_ supplements (i.e., >10,000 mcg/day). Based on the inclusion criteria, our analytic sample included 728 participants with a median age of 58 years (range 29 to 94 years), 65.4% female, and 50.3% white.

### 2.2. Blood Vitamin B_12_ Measurement

Serum holoTC was measured by an enzyme-immunoassay (EIA) for the quantitative determination of holoTC, an active form of B_12_ in human serum (Axis-Shield Diagnostics Limited, Dundee, UK, 2011) [12]. Serum vitamin B_12_ was measured by a microplate enzyme immunoassay for quantitative determination of vitamin B_12_ in human serum (Accu Bind Elisa Microwells, Monobind Inc., Lake Forest, CA, USA) [13].

### 2.3. Assessment of Dietary Intake

Diet was assessed using 24 h dietary recalls (24 HDR) and a previously validated food frequency questionnaire (FFQ) [11]. Intake of vitamin B_12_ was estimated from two sets of three 24HDRs, which included information on all foods, beverages, and supplements consumed by each subject during the previous 24 h. The first set of recalls consisted of one Saturday, one Sunday, and one weekday. The second set of recalls with the same intake days was repeated approximately six months later. For each set of recalls, we calculated weighted mean vitamin B_12_ intake as (Saturday intake + Sunday intake + 5 × weekday intake)/7 [11], and averaged these two weeks to estimate the mean daily vitamin B_12_ intake.

A trained dietitian collected the 24 h dietary recalls, using standard probes and a multiple-pass approach methodology [11]. Recall data were entered using the Nutrition Data System for Research version 4.06 or 5.0 (NDS-R, Nutrition Coordinating Center, Minneapolis, MN, USA); the analytic data were based on the NDS-R 2008 database. Vegetarian patterns were determined from FFQ according to the frequency of intake of animal foods, which includes meats (red meat + poultry), fish, and dairy (dairy + eggs) [14]. Non-vegetarians consumed meat and fish >1 per week, and no limits on dairy. Semi-vegetarians consumed some meat (once per month to once per week), and combined fish and meat 1/month to <1/week, and no limits on dairy. Pesco-vegetarians ate fish at least 1/month but meat never or rarely, and no limits on dairy. Lacto-ovo-vegetarians never or rarely ate meat and fish, and no restrictions on dairy. Vegans never or rarely ate meat, fish, and dairy.

### 2.4. Statistical Analysis

We used SPSS version 22 for all statistical analyses (IBM SPSS, Inc. Armonk, NY, USA) and we considered a *p*-value of 0.05 or less as statistically significant. Dietary sources of vitamin B_12_ were classified as supplements, animal source foods, and fortified foods (Table 1). Total B_12_ intake was the sum of B_12_ from animal food sources, fortified foods, and supplements.

For preliminary analysis, Pearson’s correlation coefficient showed significantly (*p* < 0.001) positive correlations between holoTC and serum vitamin B_12_ (*r* = 0.47), holoTC, and total vitamin B_12_ intake (*r* = 0.32), and serum vitamin B_12_ and total vitamin B_12_ intake (*r* = 0.28). Meanwhile, Spearman’s correlation coefficient showed significantly (*p* < 0.001) positive correlations between serum vitamin B_12_ and vitamin B_12_ intake from supplements (*r* = 0.30), and holoTC and vitamin B_12_ intake from supplements (*r* = 0.33) (data not shown).

Descriptive statistics using one way ANOVA, Kruskal Wallis, independent *t*-tests, or the Mann-Whitney procedure were used to test the difference in mean intake of vitamin B_12_, holoTC, and serum vitamin B_12_ by subjects’ characteristics (Table 2 and Table 3). Dietary intake of vitamin B_12_ in Table 3 was not energy-adjusted to show the real intake of subjects. 

Intake of vitamin B_12_ from fortified and animal food sources, but not supplements, were first energy-adjusted using the residual method [15]. Then vitamin B_12_ from all sources was divided into tertiles. As continuous variables, HoloTC and serum vitamin B_12_ were log transformed to approximate normality. Users and non-users of vitamin B_12_-containing supplements were identified according to their intake of vitamin B_12_ from individual vitamin B_12_ supplements and multivitamins from dietary recalls. Besides five levels of dietary patterns as noted below, two reduced levels of dietary patterns were developed: Vegetarians were a combination of vegans and lacto-ovo-vegetarians, and non-vegetarians were the combination of the remaining three patterns.

We used multivariable linear regressions to assess which dietary sources of vitamin B12 were associated with serum vitamin B_12_ and holoTC while controlling for potential confounding. The final statistical model included all together the individual exposures (B_12_ supplements, milk substitutes, cereals, meat analogs, other fortified foods, meat, fish, milk, eggs, milk and eggs), serum vitamin B_12_ or holoTC concentration as the outcome, adjusted for age, gender, race, BMI, and serum creatinine.

## 3. Results

### 3.1. Subject Characteristics and Plasma Vitamin B_12_ Concentration

Characteristics of the study population are shown in Table 2. Mean holoTC concentration among 728 subjects was 103.35 pmol/L, which significantly increased with age. HoloTC was significantly (*p* = 0.014) lower among younger males (post-hoc test showed significantly lower holoTC among 40–49 year olds compared to 70–94 year olds), and white subjects who did not use vitamin B_12_-containing supplements. Mean serum vitamin B_12_ was significantly lower among males compared to females (*p* = 0.035) and subjects who did not use vitamin B_12_-containing supplements, compared to users (*p* < 0.001). We found no significant difference of holoTC and serum vitamin B_12_ concentrations according to BMI, alcohol users, or smoking status (Table 2).

### 3.2. Dietary Sources of Vitamin B_12_ Intake

The mean total vitamin B_12_ intake from different sources is presented in Table 3. The highest intake of vitamin B_12_ came from vitamin B_12_ supplements (mean 50.34 mcg/day; median 2.14 mcg/day), followed by fortified foods (mean 3.58 mcg/day; median 1.64 mcg/day), and animal source foods (mean 1.96 mcg/day; median 1.36 mcg/day). Only 59 subjects (8.1%) had eaten nutritional yeast alone. We found that the highest sources of vitamin B_12_ intake from animal source foods came from foods made from milk and eggs (mean 1.11 mcg/day), followed by milk alone (mean 0.39 mcg/day), fish (mean 0.24 mcg/day), and meat (mean 0.18 mcg/day). Foods that contained milk, eggs, cheese, and butter were grouped with dairy and egg foods. These foods included bread, sweet bread, dessert, pudding, snacks, cookies, and cakes. The high intake of foods made from dairy and eggs might explain the low intake of eggs by itself.

The mean intake of vitamin B_12_ from different sources across age, gender, race, dietary patterns, and the use of vitamin B_12_-containing supplements is presented in Table 3. Among this population, animal source foods were significantly different (*p* = 0.004) across the age categories. Younger individuals had significantly higher intake of animal source foods than older subjects. Older subjects had significantly higher intake of vitamin B_12_ containing supplements than the younger subjects (*p* = 0.012). Females had significantly higher intake of fortified food than males (3.91 mcg/day vs. 2.95 mcg/day) at *p* < 0.001. Females also consumed more vitamin B_12_ from supplements (*p* = 0.001). White subjects had significantly higher intake of fortified food than non-white subjects (4.83 mcg/day vs 2.31 mcg/day). They also consumed more vitamin B_12_ from supplements (*p* = 0.003). Across the dietary patterns, vegans, lacto-ovo-, pesco-, and semi-vegetarians had significantly higher vitamin B_12_ intake from fortified foods than non-vegetarians. Conversely, non-vegetarians had significantly higher vitamin B_12_ intake from animal source foods than the vegetarian group (*p* < 0.001). We also found a significant difference in vitamin B_12_ intake from meat (*p* = 0.004), fish (*p* = 0.008), and foods made from milk and eggs (*p* = 0.002) within the age categories, with B_12_ intake from these foods inversely associated with age, in general. Non-white subjects had significantly higher vitamin B_12_ intake from meat (0.24 mcg/day vs. 0.12 mcg/day) and fish (0.36 mcg/day vs. 0.12 mcg/day) than white subjects. On the other hand, white subjects had significantly higher vitamin B_12_ intake from milk than non-white subjects (0.51 mcg/day vs. 0.26 mcg/day). Furthermore, there were significant differences of vitamin B_12_ intake from meat, fish, milk, and eggs based on dietary patterns. As expected, non-vegetarian subjects had higher vitamin B_12_ intake from meat than vegan, lacto-ovo-, pesco-, or semi-vegetarian subjects. Pesco-vegetarians and non-vegetarians had higher fish intake than other vegetarians.

### 3.3. Dietary Sources of Vitamin B_12_ and Vitamin B_12_ Status

Table 4 shows the models of dietary sources of vitamin B_12_ associated with holoTC and serum vitamin B_12_ adjusted for all B_12_ intake from different sources, age, gender, race, BMI, and serum creatinine in vegetarians and non-vegetarians. In general, higher compared to lower intakes of B_12_ supplements were significantly associated with higher concentration of each biomarker in vegetarians and non-vegetarians.

Compared to non-consumers, intake of B_12_ from fish was significantly inversely associated with holoTC, and the lowest compared to moderate intake of B_12_ from other fortified foods was significantly inversely associated with serum B_12_ in vegans and lacto-ovo-vegetarians. This significant inverse association disappeared when we stratified analysis based on supplement users (data not shown). In all subjects and non-vegetarians, vitamin B_12_ from milk substitutes was significantly associated with holoTC, followed by B_12_ from eggs with holoTC and B_12_ from meat and milk with serum B_12_ in non-vegetarians.

Table 5 presents the regression model stratified on the use of vitamin B_12_-containing supplements. Among non-users of vitamin B_12_ supplements, intake of B_12_ from milk substitutes in the upper compared to the lower tertile was significantly associated with holoTC and serum vitamin B_12._ Among the users of vitamin B_12_-containing supplements, compared to the lowest tertile, we also found a positive association between vitamin B_12_ from meat in the middle tertile and serum vitamin B_12_.

## 4. Discussion

Several findings from this study have important implications for this population. Firstly, supplements are a major source of vitamin B_12_. Secondly, a vegetarian diet plays an important role in vitamin B_12_ intake. These findings were not surprising, since the majority of subjects in the present study were vegetarians who had limited sources of vitamin B_12_ from animal source foods. This study found intake of B_12_ from animal source foods was significantly higher among non-vegetarians. On the other hand, B_12_ intake from fortified foods was significantly higher among vegans and lacto-ovo-vegetarians, particularly among vegans and lacto-ovo-vegetarians who did not use B_12_ supplements. Nearly 60% of subjects in AHS-2 used vitamin B_12_ supplements, while in other studies, 19–59% of vegans and 4.4–20% of vegetarians (lacto-ovo- and lacto-vegetarians) use vitamin B supplements [16,17,18]. More recently, Sobiecki [19] showed that 50.1% vegans and 38.7% vegetarians used at least one supplement as a source of vitamin B_12_. The mean total intake of vitamin B_12_ by source in this present study (total 55.8 mcg/day, vitamin B_12_ supplement 50.34 mcg/day; all fortified foods 3.58 mcg/day; and animal source foods 1.96 mcg/day) was higher than the mean total vitamin B12 intake among a sample of Americans in the Framingham Offspring Study (total 8.7 mcg/day, 15.3 mcg/day for supplement users, and 6.2 mcg/day for non-supplement-users) [7], and also among the study population of the Hordaland Homocysteine Study (6 mcg/day) [8].

Although not all subjects in the current study obtained vitamin B_12_ from supplements, multiple linear regressions showed that supplements were the strongest predictor of holoTC and serum vitamin B_12_, independent of other dietary sources of B_12_, age, gender, race, BMI, and serum creatinine. This supports findings from the Framingham Offspring Study on the importance of vitamin B_12_ supplements in maintaining normal plasma vitamin B_12_ concentrations [7]. By comparison, the mean B_12_ plasma concentration was lower (265 ± 150.97 pmol/L) in the present study than the Framingham cohort 329 ± 3.1 pmol/L. Further questions relate to whether there is a dose response of vitamin B_12_ intake from supplements in maintaining plasma vitamin B_12_ concentration in this population. Study results showed increased intake of B_12_ from supplements significantly increased holoTC and serum B_12_ in vegans and lacto-ovo-vegetarians (*p* trend < 0.001), and in non-vegetarians (*p* trend < 0.001).

On stratification by B_12_ supplement use, our findings suggest that among non-users of B_12_ supplements, milk substitutes in particular were strong predictors of holoTC and serum vitamin B_12_. This could be due to the consumption of fortified soy milk, rice milk, and almond milk, which are popular in this AHS-2 sample. In the Framingham Offspring Study, fortified cereals were significantly associated with plasma vitamin B_12_ [7]. The importance of supplements and fortified foods as reliable sources of vitamin B_12_ among vegans also support the statement of Academy of Nutrition and Dietetics [20], which indicate that varieties of milk substitutes have similar vitamin B_12_ content based on the food label. However, for alternate dairy beverages, the nutrient labels should be read carefully because not all soy milk, almond milk, and rice milk are fortified with vitamin B_12_.

Besides B_12_ supplements and fortified foods, animal-sources foods are still important as vitamin B_12_ sources, particularly among non-vegetarians. Intake of B_12_ from meat and milk have an effect on serum B_12_, while intake of B_12_ from eggs has an effect on holoTC among non-vegetarians. Furthermore, among B_12_ supplement users, B_12_ intake from meat, particularly in the middle intake tertile, has effects on B_12_ status. However, mean B_12_ intake from all animal sources in the AHS-2 (1.96 mcg/day) was lower compared to the general population. For example, mean intake of vitamin B_12_ from dairy foods among a sample of Americans ranged from 4.5 mcg (the lowest tertile) to 8.2 mcg/day (upper tertile), and the mean intake of vitamin B_12_ from meat was in the range 3.6 mcg/day (the lowest tertile) to 10.1 mcg/day (the upper tertile) [7]. Further, vitamin B_12_ intake from animal sources was also lower than vitamin B_12_ intake from foods among healthy older Dutch adults, in whom the mean intake was 3.41 mcg for women and 4.5 mcg for men [9]. In AHS-2, mean B_12_ intake from meat was 0.18 mcg/day, from fish was 0.24 mcg/day, from milk was 0.39 mcg/day, and from milk and eggs was 1.11 mcg/day.

A striking finding from the current study is that mean intake of B_12_ from animal source foods in all dietary groups, except for non-vegetarians, and also in the various age categories, were below the recommended dietary allowance (RDA) of 2.4 mcg/d for adults [21]. However, intake from fortified foods, except for non-vegetarians, exceeded the RDA. Because a substantial proportion of elderly individuals show malabsorption of protein-bound B_12_ from animal source foods, the RDA recommends individuals 51 years of age or older regularly use B_12_ fortified foods and supplements. In this primarily older cohort, mean daily intake of B_12_ from fortified food and supplements was 3.6 mcg and 50.3 mcg, respectively, and mean daily intake from supplements increased with increasing age (*p* < 0.012). These results suggest that AHS-2 participants were compliant with guidelines on B_12_ fortified food and supplement use. Whether the consistent consumption of B_12_ fortified foods alone, or the additional intake of supplements, is needed to support B_12_ status in individuals adhering to dietary patterns devoid or low in animal source foods requires further study. 

As with others, our study has its strengths and weaknesses. One strength is that we included vegetarians and non-vegetarians who were randomly selected and were representative of the larger parent AHS-2 cohort population, while a previously published report was conducted in a macrobiotic community [18]. The use of multiple 24 h dietary recalls is valuable as it incorporates day-to-day variation in the diet, which is important in estimating usual intake of vitamin B_12_ and other micronutrients [22]. The main limitation of this study is its cross-sectional approach, which cannot determine causal effects. The subjects with missing data of interest were significantly different by race (*p* < 0.001), but not by age and gender. Although the sample was spread geographically throughout the U.S. and Canada, these results might not be considered as fully representative of the general US population.

## 5. Conclusions

In conclusion, supplements containing vitamin B_12_ were significant contributors of plasma vitamin B_12_ concentrations in this population, particularly among vegan and lacto-ovo-vegetarians, followed by B_12_ from milk substitutes among non-users of B_12_-containing supplements. Among non-users of supplements, milk substitutes were important contributors to plasma B_12_ concentrations. Among vegan and lacto-ovo-vegetarians, vitamin B_12_ from supplements was associated with plasma vitamin B_12_, as were milk substitutes, meat, milk, and eggs among non-vegetarians. This information may be used in planning nutritionally adequate diets among vegetarians and non-vegetarians, and in designing future clinical trials to examine which B_12_ dietary sources have the potential to prevent B_12_ deficiency among non-users of B_12_ supplements.

## Figures and Tables

**Table 1:** Dietary Sources of Vitamin B_12_ in the Adventist Health Study-2 (AHS-2) Calibration Study.

Food Classification	Dietary Sources
Animal-sources foods	Meat: beef, lamb, goat, pork, poultry
	Fish: fish, seafood
	Milk: milk, yogurt, cheese, ice cream, cream
	Eggs
	Food made with milk and eggs: pudding, dessert, cakes, cookies
B12 fortified foods	Cereals
	Meat analogs
	Milk substitute: soy milk, rice milk, almond milk
	Brewer’s yeast, torula yeast
	Other fortified foods include vegetarian foods, energy drinks
Vitamin B12 containing supplement	Individual vitamin B_12_
	Multi-vitamin containing vitamin B_12_

**Table 2:** Characteristics of the Study Population and Plasma Concentration of Vitamin B_12_.

	*n*	%	HoloTC (pmol/L)		Serum Vitamin B_12_ (pmol/L)	
			Mean ± SD	*p* Value	Mean ± SD	*p* Value
**All**	728	100	103.35 ± 53.17		265.00 ± 150.97	
**Age (years)**						
29–39	56	7.6	93.55 ± 42.15	0.014	244.47 ± 75.58	0.232
40–49	154	21.2	93.11 ± 42.81		249.83 ± 121.34	
50–59	192	26.4	104.00 ± 57.30		272.35 ± 165.79	
60–69	165	22.7	107.44 ± 55.29		259.80 ± 130.60	
70–94	161	22.1	111.57 ± 56.65		283.23 ± 190.87	
**Gender**						
Male	252	34.6	94.63 ± 51.03	0.001	248.73 ± 152.20	0.035
Female	476	65.4	107.96 ± 53.75		273.61 ± 149.76	
**Race**						
White	366	50.3	98.90 ± 53.63	0.023	264.66 ± 176.73	0.952
Non-white	362	49.7	107.84 ± 52.39		265.34 ± 119.68	
**Dietary patterns**						
Vegan	67	9.2	107.53 ± 59.67	0.033	292.26 ± 214.52	0.052
Lacto-ovo	207	28.4	109.86 ± 57.61		275.32 ± 164.40	
Pesco	78	10.7	111.10 ± 48.43		277.59 ± 129.10	
Semi	35	4.8	90.92 ± 41.05		211.06 ± 66.39	
Non-vegetarian	341	46.8	98.07 ± 50.56		256.05 ± 136.33	
**B_12_ Containing Supplement**						
User	435	59.8	114.09 ± 54.57	<0.001	281.95 ± 161.89	<0.001
Non-user	293	40.2	87.40 ± 46.74		239.85 ± 129.36	
**Body Mass Index ^a^**						
<25	254	34.9	106.88 ± 56.35	0.148	265.90 ± 160.34	0.088
25–29.9	244	33.5	104.78 ± 52.04		276.82 ± 164.96	
≥30	209	28.7	97.58 ± 48.54		246.34 ± 103.53	
**Alcohol user ^a^**						
Never	421	57.8	105.60 ± 53.24	0.148	266.58 ± 159.01	0.731
Ever	295	40.5	99.81 ± 51.94		262.62 ± 140.33	
**Smoking**						
Never	604	83	104.09 ± 52.70	0.402	264.63 ± 146.51	0.881
Ever	124	17	99.70 ± 55.49		266.85 ± 171.69	

^a^ The proportion of data missing was 2.9% of BMI and 1.6% of alcohol users.

**Table 3:** Mean and Standard Deviation of Vitamin B_12_ intake (mcg/day) from Different Sources by Subject Characteristic ^†^.

	*n*	B_12_ from Animal Source Foods						B_12_ Fortified Foods					B_12_ Containing Supplements	Total/Day
		Meat	Fish	Milk	Eggs	Milk & Eggs	Sub Total	Cereal	Meat Analog	Milk Substitutes	Other Fortified Food	Sub Total		
**Total/day**	728	0.18 ± 0.47	0.24 ± 0.88	0.39 ± 0.60	0.02 ± 0.11	1.11 ± 1.38	1.96 ± 1.95	0.73 ± 1.20	0.04 ± 0.21	0.54 ± 1.00	2.25 ± 16.3	3.58 ± 16.55	50.34 ± 178.73	55.8 ± 182.3
**Age (year)**														
29–39	56	0.21 ± 0.4	0.08 ± 0.3	0.45 ± 0.5	0.02 ± 0.1	1.26 ± 1.2	2.04 ± 1.6	0.57 ± 0.8	0.04 ± 0.2	0.46 ± 0.9	2.42 ± 7.8	3.51 ± 8.6	16.0 ± 39.9	21.5 ± 40.4
40–49	154	0.23 ± 0.5	0.23 ± 0.7	0.46 ± 0.7	0.03 ± 0.1	1.19 ± 1.3	2.16 ± 1.9	0.67 ± 1.1	0.06 ± 0.2	0.47 ± 0.9	1.30 ± 3.7	2.51 ± 4.05	37.9 ± 165.8	42.6 ± 166.3
50–59	192	0.15 ± 0.3	0.29 ± 1.2	0.40 ± 0.5	0.02 ± 0.08	1.12 ± 1.4	2.00 ± 2.2	0.84 ± 1.4	0.05 ± 0.2	0.52 ± 0.9	1.24 ± 2.4	2.67 ± 3.1	59.3 ± 230.9	64.0 ± 231.0
60–69	165	0.21 ± 0.6	0.34 ± 0.8	0.32 ± 0.4	0.03 ± 0.1	1.15 ± 1.4	2.08 ± 1.9	0.63 ± 1.0	0.02 ± 0.1	0.64 ± 1.1	5.14 ± 33.3	6.45 ± 33.6	50.3 ± 156.1	58.9 ± 172.8
70–94	161	0.14 ± 0.3	0.13 ± 0.4	0.35 ± 0.5	0.02 ± 0.08	0.92 ± 1.3	1.58 ± 1.7	0.82 ± 1.2	0.02 ± 0.09	0.58 ± 0.9	1.33 ± 4.3	2.75 ± 4.7	63.3 ± 169.9	67.7 ± 170.1
*p*-value		0.004	0.008	0.052	0.395	0.002	0.004	0.426	0.557	0.012	0.695	0.63	0.012	0.078
**Gender**														
Male	252	0.23 ± 0.6	0.31 ± 1.1	0.39 ± 0.6	0.03 ± 0.1	1.18 ± 1.5	2.16 ± 2.3	0.99 ± 1.4	0.05 ± 0.2	0.55 ± 0.9	1.35 ± 3.0	2.95 ± 3.8	35.4 ± 146.3	40.5 ± 146.5
Female	476	0.16 ± 0.3	0.20 ± 0.6	0.39 ± 0.6	0.02 ± 0.09	1.07 ± 1.2	1.86 ± 1.7	0.59 ± 1.0	0.03 ± 0.1	0.54 ± 1.0	2.73 ± 20.0	3.91 ± 20.2	58.2 ± 193.3	64.0 ± 198.4
*p*-value		0.846	0.678	0.481	0.047	0.66	0.379	<0.001	0.878	0.252	0.008	<0.001	0.001	0.261
**Race**														
White	366	0.12 ± 0.3	0.12 ± 0.8	0.51 ± 0.6	0.03 ± 0.1	1.15 ± 1.3	1.95 ± 1.9	0.92 ± 1.3	0.02 ± 0.1	0.63 ± 1.1	3.24 ± 22.6	4.83 ± 22.8	54.5 ± 172.0	61.3 ± 179.1
Non-white	362	0.24 ± 0.5	0.36 ± 0.9	0.26 ± 0.4	0.02 ± 0.1	1.07 ± 1.3	1.97 ± 1.9	0.54 ± 1.0	0.06 ± 0.2	0.46 ±0.8	1.24 ± 4.1	2.31 ± 4.6	46.1 ± 185.3	50.4 ± 185.6
*p*-value		<0.001	<0.001	<0.001	0.59	0.22	0.897	<0.001	0.539	0.052	0.002	<0.001	0.003	<0.001
**Dietary patterns**														
Vegan	67	<0.01 ± 0.01	0.07 ± 0.3	0.01 ± 0.05	0.006 ± 0.04	0.35 ± 0.7	0.46 ± 0.8	0.68 ± 1.1	0.07 ± 0.2	0.92 ± 1.2	3.10 ± 14.5	4.78 ± 15.1	96.0 ± 196.2	101.2 ± 202.9
Lacto-ovo	207	0.01 ± 0.09	0.02 ± 0.1	0.33 ± 0.5	0.01 ± 0.07	0.92 ± 1.2	1.31 ± 1.4	0.89 ± 1.3	0.06 ± 0.2	0.79 ± 1.2	4.58 ± 28.9	6.34 ± 29.0	60.5 ± 172.7	68.1 ± 182.5
Pesco	78	0.05 ± 0.1	0.65 ± 1.0	0.19 ± 0.2	0.02 ± 0.06	1.18 ± 1.7	2.11 ± 2.0	0.63 ± 1.1	0.06 ± 0.3	0.63 ± 0.9	1.88 ± 5.3	3.21 ± 5.3	27.8 ± 115.3	33.1 ± 115.6
Semi	35	0.17 ± 0.3	0.004 ± 0.02	0.64 ± 0.8	0.005 ± 0.01	1.01 ± 1.4	1.83 ± 1.6	1.00 ± 1.4	0.01 ± 0.04	0.35 ± 0.8	2.56 ± 6.6	3.93 ± 7.6	14.8 ± 25.2	20.5 ± 27.4
Non-veg	341	0.36 ± 0.6	0.34 ± 1.1	0.52 ± 0.6	0.04 ± 0.1	1.37 ± 1.4	2.64 ± 2.0	0.64 ± 1.1	0.02 ± 0.1	0.33 ± 0.6	0.71 ± 2.5	1.71 ± 3.0	43.9 ± 197.2	48.3 ± 197.5
*p*-value		<0.001	<0.001	<0.001	0.003	<0.001	<0.001	0.014	<0.001	<0.001	<0.001	<0.001	0.073	0.761

^†^ Not energy-adjusted; comparisons were made using Mann-Whitney or Kruskal Wallis.

**Table 4:** Associations of Dietary Sources of Vitamin B_12_ with Serum Vitamin B_12_ and holotranscobalamin (HoloTC) Concentration among Vegetarians (*n* = 274) and Non-vegetarians (*n* = 454) from Multivariable Regression Analysis ^a^.

	Serum Vitamin B_12_		HoloTC	
	Vegetarians	Non-Vegetarians	Vegetarians	Non-Vegetarians
	Coef. (95% CI)	Coef. (95% CI)	Coef. (95% CI)	Coef. (95% CI)
**B_12_ supplement**				
Non-user (0)	Ref.	Ref.	Ref.	Ref.
0.001–8.929 mcg/day	−22.1 (−79.5, 35.0)	19.3 (−10.3, 49.1)	3.9 (−14.4, 22.4)	18.2 (7.5, 28.9) ^‡^
≥8.930 mcg/day	88.7 (38.9, 138.6) ^‡^	61.2 (31.7, 90.6) ^‡^	30.1 (14.0, 46.2) ^‡^	37.0 (26.4, 47.6) ^‡^
*p*-trend	<0.001	<0.001	<0.001	<0.001
**Milk substitutes**				
Non-consumer (0)	Ref.	Ref.	Ref.	Ref.
0.01–0.5 mcg/day	−37.2 (−102.1, 27.7)	19.6 (−17.8, 57.1)	6.4 (−14.5, 27.4)	2.8 (−10.5,16.3)
≥0.51 mcg/day	37.3 (−16.1, 90.8)	9.6 (−21.2, 40.6)	8.0 (−9.1, 25.3)	15.6 (4.5, 26.7) ^†^
*p*-trend	0.12	0.54	0.36	0.01
**Cereals**				
Non-consumer (0)	Ref.	Ref.	Ref.	Ref.
0.01–0.61 mcg/day	19.4 (−38.7, 77.5)	15.5 (−15.0, 46.2)	−5.4 (−24.1, 13.3)	−7.6 (−18.6, 3.3)
≥0.62 mcg/day	−46.6 (−103.4, 10.1)	16.6 (−14.6, 47.9)	−6.1 (−24.5, 12.1)	−1.5 (−12.7, 9.7)
*p*-trend	0.12	0.14	0.45	0.88
**Meat analogs**				
Non-consumer (0)	Ref.	Ref.	Ref.	Ref.
≥0.01 mcg/day	−4.6 (−57.3, 48.1)	−22.3 (−71.9, 27.1)	−1.9 (−18.9, 15.1)	−13.6 (−31.4, 4.2)
**Other fortified foods**				
≤0.42 mcg/day	Ref.	Ref.	Ref.	Ref.
0.43–0.69 mcg/day	−64.1 (−115.3, −2.8) ^†^	−3.8 (−32.5, 24.8)	−2.9 (−22.7, 16.8)	−0.3 (−10.7, 9.9)
≥0.70 mcg/day	−8.9 (−68.7, 50.8)	5.4 (−26.7, 37.7)	0.8 (−18.4, 20.1)	−0.4 (−11.6, 11.5)
*p*-trend	0.65	0.78	0.73	0.95
**Meat**				
Non-consumer (0)	Ref.	Ref.	Ref.	Ref.
0.01–0.40 mcg/day	−38.1 (−171.0, 94.6)	46.9 (11.0, 82.9) ^†^	−21.5 (−64.4, 21.3)	−6.8 (−19.7, 6.0)
≥0.41 mcg/day	−11.7 (−161.5, 138.1)	5.4 (−26.5, 37.4)	32.9 (−15.4, 81.2)	−10.3 (−21.8, 1.1)
*p*-trend	0.66	0.75	0.49	0.07
**Fish**				
Non-consumer (0)	Ref.	Ref.	Ref.	Ref.
≥0.01 mcg/day	−37.1 (−131.4, 57.1)	11.1 (−14.5, 36.7)	−36.3 (−66.8, −5.9) ^†^	0.1 (−9.0, 9.3)
**Milk**				
≤0.39 mcg/day	Ref.	Ref.	Ref.	Ref.
0.40–0.51 mcg/day	−10.3 (−64.0, 43.2)	7.6 (−26.1, 41.3)	−2.5 (−19.8,14.7)	−5.2 (−17.2, 6.6)
≥0.51 mcg/day	0.9 (−61.7, 63.6)	33.4 (0.6, 66.2) ^†^	−1.5 (−21.7, 18.7)	0.8 (−11.9, 13.6)
*p*-trend	0.93	0.53	0.64	0.86
**Eggs**				
Non-consumer (0)	Ref.	Ref.	Ref.	Ref.
≥0.01 mcg/day	−55.9 (−118.6, 6.8)	−8.3 (−38.3,21.7)	−3.8 (−24.1, 16.4)	10.9 (0.1, 21.8) ^†^
**Milk and eggs**				
≤0.51 mcg/day	Ref.	Ref.	Ref.	Ref.
0.52–0.74 mcg/day	−13.7 (−63.4, 36.0)	4.2 (−28.7,37.3)	−8.2 (−24.3, 7.7)	4.0 (−8.0, 16.1)
≥0.75 mcg/day	−22.6 (−84.3, 39.0)	30.1 (−1.6,61.9)	8.3 (−11.5, 28.2)	5.7 (−6.0, 17.5)
*p*-trend	0.55	0.03	0.51	0.48

Ref.: Represents the comparison group in regression analysis. ^†^ *p* ≤ 0.05; ^‡^ ≤ 0.001; ^a^ The model included all the variables in this table together adjusted for age, gender, race, BMI, and serum creatinine.

**Table 5:** Associations of Dietary Sources of Vitamin B_12_ with Serum Vitamin B_12_ and HoloTC Concentration among Users (*n* = 435) and Non-Users (*n* = 293) of Vitamin B_12_-containing Supplements from Multivariable Regression Analysis ^a^.

	Serum Vitamin B_12_		HoloTC	
	User	Non-User	User	Non-User
	Coef. (95% CI)	Coef. (95% CI)	Coef. (95% CI)	Coef. (95% CI)
**Milk substitutes**				
Non-consumer (0)	Ref.	Ref.	Ref.	Ref.
0.01–0.5 mcg/day	−12.0 (−58.4, 34.3)	−21.2 (−70.7, 28.2)	2.4 (−12.8, 17.8)	1.4 (−15.9, 18.7)
≥0.51 mcg/day	14.8 (−24.4, 54.1)	47.2 (8.02, 86.4) ^†^	8.7 (−4.2, 21.7)	20.3 (6.5, 34.0) ^†^
*p*-trend	0.60	0.03	0.24	0.004
**Cereals**				
Non-consumer (0)	Ref.	Ref.	Ref.	Ref.
0.01–0.61 mcg/day	25.0 (−14.5, 64.5)	15.1 (−25.9, 56.3)	−8.7 (−21.8, 4.3)	1.1 (−13.3, 15.5)
≥0.62 mcg/day	−6.3 (−46.5, 33.8)	2.0 (−37.7, 41.9)	−5.8 (−19.2, 7.4)	−2.8 (−16.7, 11.1)
*p*-trend	0.77	0.96	0.63	0.79
**Meat analogs**				
Non-consumer (0)	Ref.	Ref.	Ref.	Ref.
≥0.01 mcg/day	−4.0 (−48.4, 40.3)	−23.6 (−84.3, 36.9)	−9.7 (−24.4, 4.9)	4.2 (−16.9, 25.5)
**Other fortified foods**				
≤0.42 mcg/day	Ref.	Ref.	Ref.	Ref.
0.43–0.69 mcg/day	−34.4 (−74.0, 5.09)	−6.8 (−45.6, 32.0)	−8.4 (−21.5, 4.6)	6.9 (−6.6, 20.5)
≥0.70 mcg/day	7.9 (−34.0, 49.9)	3.8 (−35.5, 43.2)	4.0 (−9.9, 17.9)	−2.1 (−15.9, 11.6)
*p*-trend	0.69	0.99	0.48	0.71
**Meat**				
Non-consumer (0)	Ref.	Ref.	Ref.	Ref.
0.01–0.40 mcg/day	59.2 (8.4, 110.0) ^†^	6.8 (−53.3, 39.6)	1.7 (−15.0, 18.5)	−10.9 (−27.2, 5.3)
≥0.41 mcg/day	−12.1 (−56.7, 32.4)	−9.7 (−50.2, 30.7)	−11.5 (−26.3, 3.1)	−4.5 (−18.7, 9.6)
*p*-trend	0.58	0.67	0. 099	0.53
**Fish**				
Non-consumer (0)	Ref.	Ref.	Ref.	Ref.
≥0.01 mcg/day	−4.2 (−43.2, 34.7)	2.6 (−34.4, 39.7)	−6.1 (−19.1, 6.7)	−3.2 (−16.2, 9.7)
**Milk**				
≤0.39 mcg/day	Ref.	Ref.	Ref.	Ref.
0.40–0.51 mcg/day	−35.8 (−77.1, 5.3)	−3.5 (−42.4, 35.5)	−9.0 (−22.6, 4.6)	−0.9 (−14.5, 12.7)
≥0.51 mcg/day	−12.8 (−58.2, 32.5)	−5.0 (−48.5, 38.5)	−3.0 (−18.1, 11.9)	3.9 (−11.2, 19.2)
*p*-trend	0.63	0.75	0.91	0.82
**Eggs**				
Non-consumer (0)	Ref.	Ref.	Ref.	Ref.
≥0.01 mcg/day	−27.8 (−68.6, 12.8)	0.96 (−41.1, 43.0)	4.7 (−8.7, 18.2)	7.3 (−7.3, 22.1)
**Milk and eggs**				
≤0.51 mcg/day	Ref.	Ref.	Ref.	Ref.
0.52–0.74 mcg/day	−23.0 (−64.4, 18.2)	10.3 (−28.3, 49.1)	−1.7 (−15.4, 11.9)	−3.0 (−16.6, 10.5)
≥0.75 mcg/day	1.4 (−39.7, 42.6)	24.2 (−18.2, 66.6)	4.0 (−9.5, 17.7)	2.5 (−12.3, 17.4)
*p*-trend	0.70	0.32	0.50	0.78

Ref.: Represents the comparison group in regression analysis. ^†^ *p* ≤ 0.05; ^a^ All variables were included together and adjusted for age, gender, race, BMI, and serum creatinine.
